# Endometrial microbiota profile in in-vitro fertilization (IVF) patients by culturomics-based analysis

**DOI:** 10.3389/fendo.2023.1204729

**Published:** 2023-08-04

**Authors:** Federica Cariati, Consolata Carotenuto, Francesca Bagnulo, Daniela Pacella, Vincenzo Marrone, Rossella Paolillo, Maria Rosaria Catania, Raffaella Di Girolamo, Alessandro Conforti, Ida Strina, Carlo Alviggi

**Affiliations:** ^1^ Department of Public Health, School of Medicine, University of Naples Federico II, Naples, Italy; ^2^ Department of Molecular Medicine and Medical Biotechnology, University of Naples Federico II, Naples, Italy; ^3^ Department of Neuroscience, Reproductive Science and Odontostomatology, University of Naples Federico II, Naples, Italy

**Keywords:** microbiota, endometrium, embryo transfer, IVF, MALDI

## Abstract

**Introduction:**

It is well recognized that the human uterus and adjoining tissues of the female reproductive tract exist in a non-sterile state where dysbiosis can impact reproductive outcomes. The endometrial microbiota is a part of this greater milieu. To date, it has largely been studied using 16S rRNA or metagenomics-based methodologies. Despite the known advantages of sequencing analysis, several difficulties have been noted including sample contamination and standardization of DNA extraction or sequencing. The aim of this study was to use a culturomics-based method to analyze the endometrial microbiota and correlate the results with ongoing pregnancy rates.

**Methods:**

A prospective cohort study was performed at the University of Naples from October 2022 and February 2023. Ninety-three patients undergoing an IVF cycle with single embryo transfer (ET) (fresh or frozen) were enrolled in the study. Following ET, the catheter tip was inserted into brain heart infusion (BHI) medium under sterile conditions for culture. After 24h and 48h of incubation the microorganisms in the colonies were identified by matrix-assisted laser desorption/ionization time-of-flight mass spectrometry (MALDI-TOF MS).

**Results:**

Overall, 68 (73,92%) patients resulted positive for one or more microbes and 25 patients (26,08%) had no microbial growth. Across all participants, the four most important phyla were *Firmicutes* (87,76%), *Proteobacteria* (27,94%), *Actinobacteria* (10,29%) and *Ascomycota* (8,82%). *Lactobacillus* species, in particular, was significantly correlated with ongoing pregnancy rate (p=0,05). On the other hand, Staphylococcus subspecies (spp.) (p<0,05) and Enterobacteriaceae (p<0,001) were found to have a negative impact on the implantation rate.

**Discussion:**

Detection of bacteria by culturomics from catheter tips used for embryo transfer has been shown to be a reliable method to detect pathogen growth. Endometrial microbiota testing in clinical practice could certainly offer a means to further improve diagnosis and treatment strategies in IVF patients.

## 1 Introduction

Human microbiota is critical to understanding human health (1). It represents the complex community of microbes that inhabit a specific site of the body and the environment itself (2). The Human Microbiome Project has revealed that approximately 9% of the total human microbiome is found in the female reproductive tract (3). The presence of different microorganisms in the female and male reproductive tracts may affect reproductive function (4). There is growing evidence to support the existence of a uterine microbiome. Uterine bacterial colonization could originate in the gut, oral cavity, bloodstream, vagina and cervical microbiota *via* ascension (5). However, its composition remains to be fully investigated. In fact, while the right balance of commensal bacteria could help maintain a eubiotic state, a dysbiotic state could be linked to pathological alteration such as pelvic inflammatory disease (PID), endometriosis, endometritis and also infertility (6–9). Definitely, bacterial vaginosis is the most well-known dysbiotic state in vagina that can also disturb the pregnancy rate and IVF success rate. This is because women with a low percentage of *Lactobacillus* in their vaginal sample appear less likely to have a successful embryo implant (10). Notwithstanding, data about the impact of *Lactobacillus* on embryo implantation is still controversial. Furthermore, despite the technological advancement in IVF of single ET and preimplantation genetic testing, the proportion of euploid embryos failing to implant is approximately 40-55%, suggesting that others factors could contribute to reproductive senescence and failure (11, 12). For these reasons there is increasing interest in endometrial receptivity and embryo implantation process study in order to increase success rates. Embryo implantation is the result of a complex interplay between embryo and uterine environment, depending also on several factors including hormonal axis and immune system. At the same time local and systemic immunity are influenced by microbiome. The identification of bacterial species has been made by Next Generation Sequencing (NGS) technology and especially by Metagenomics and the 16S gene analysis methods. On the other hand, a complementary strategy was also recently developed: culturomics. It consists of multiple culture conditions combined with the rapid identification of bacteria and allows for the culture of hundreds of new microorganisms providing exciting new perspectives in clinical practice. The advantage of culturomics is that it offers the means to analyze the complex microbial ecosystems in the uterus to detect minority populations; it is also not limited to eubacteria (12).

The primary aim of this study was to investigate the application of culturomics in clinical practice for endometrial microbiota analysis in patients undergoing IVF. The secondary aim was to correlate culturomics results with IVF outcome.

## 2 Materials and methods

Ninety-three infertile women aged between 29 and 47 years, undergoing the IVF technique between October 2022 and February 2023 at the IVF Unit, University of Naples Federico II, were included in the study. Specifically we included patients undergoing embryo transfer with a blastocyst classified as good quality based on morphology evaluation following the Gardner and Schoolcraft criteria (13). The exclusion criteria were: recent diagnosis of pelvic inflammatory disease (PID), clinically relevant abnormalities of the endometrial cavity including fibroids, endometrial polyps and septate uterus, endometrial hyperplasia or cancer, oocyte recovery failure or development of poor quality blastocytes, cervico-vaginal infections, sexually transmitted disease, antimicrobial treatment in the last 4 weeks.

Before the IVF technique, microbiological, molecular and culture screening were prescribed to all patients in the study as well as routine blood chemistry, genetic and hormonal tests. Specifically, vaginal secretions were collected from each enrolled patient to search for common germs, fungi, *Neisseria gonorrhoeae*, *Gardnerella vaginalis*, *Trichomonas vaginalis* and cervical secretions to search for *Chlamydia trachomatis*, *Mycoplasma hominis*, *Ureaplasma urealyticum/Ureaplasma parvum* within 6 months before the sampling. All patients with a positive vaginal swab were subjected to antibiotic and/or antifungal therapy by local and systemic administration. Furthermore, the only patients recruited for embryo transfer were those from whom it was possible to obtain mature oocytes and subsequently blastocyst stage embryos and/or day 3 embryos. The investigations were carried out following the rules of the Declaration of Helsinki (https://www.wma.net/what-we-do/medical-ethics/declaration-of-helsinki (accessed on 12 March 2021). A written informed consent form was signed by all participants involved in the study.

### 2.1 The sampling

The embryo transfer was performed using the double-lumen catheter set in order to avoid the contamination from cervical canal. In details, the guiding (outer) catheter with a bigger diameter allow the isolation of the transfer (inner) catheter, during the embryo transfer. The endometrial fluid, aspirated for capillarity and thanks to a syringe was added to 8mL of BHI culture medium taking great care to avoid any contact with non-sterile surfaces. The samples thus obtained were sent to the laboratory for microbiological culture investigation.

### 2.2 Culturomics

**Figure 1:**
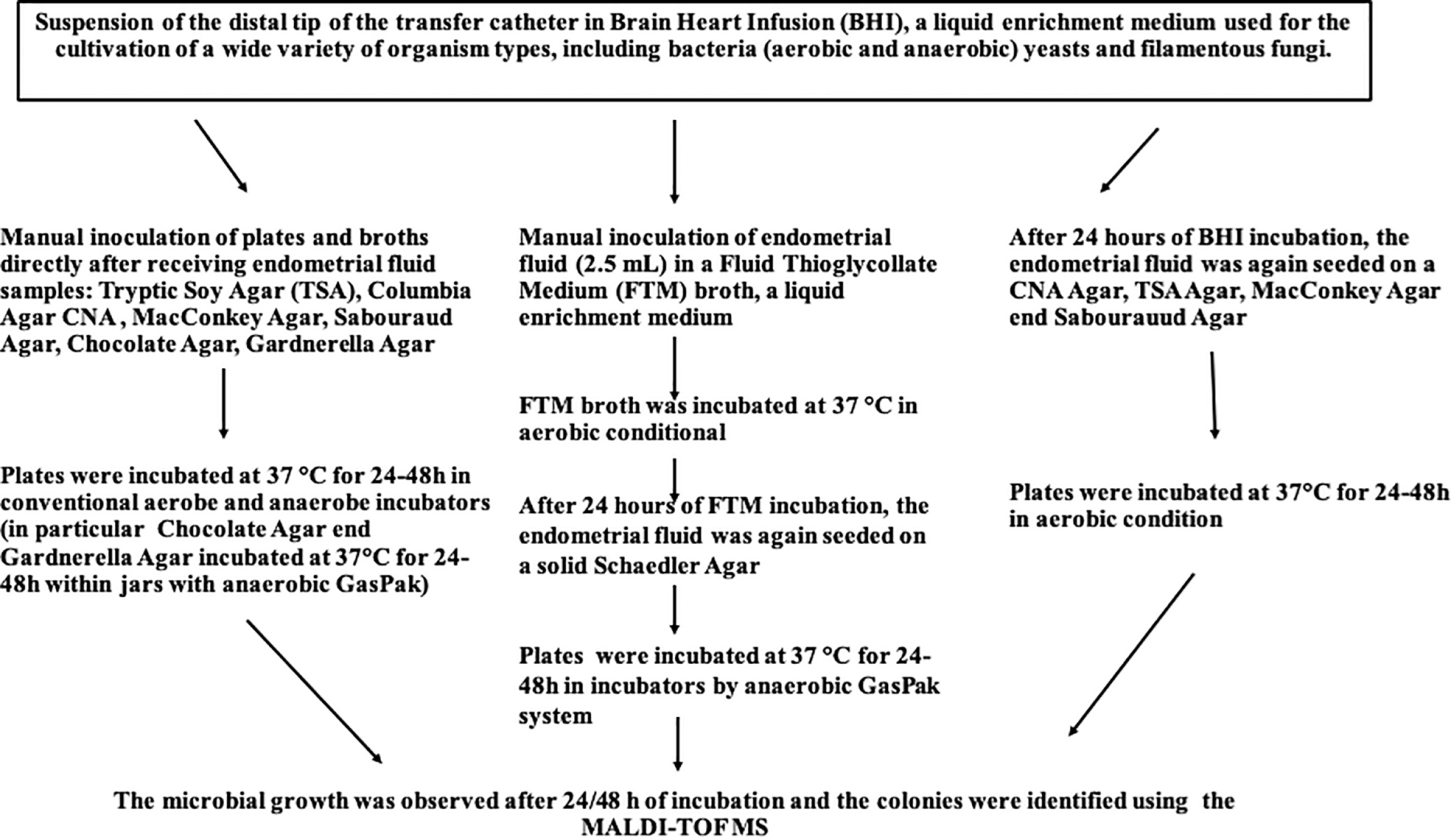
Flowchart of Culturomics-based Analysis.

Bacteriological analysis was performed in the laboratory of Bacteriology and Mycology at the Department of Molecular Medicine and Medical Biotechnology of the University of Naples Federico II (**Figure 1**). Microbial culture of the distal tip of the transfer catheter was performed to evaluate and identify the endometrial microflora at the time of ET. Immediately after performing the ET, the catheter tip was resuspended within a liquid BHI medium, highly nutritious. The samples for culture investigation were received in the laboratory within 10-15 minutes from the ET, and subsequently seeded on plates of Tryptic Soy Agar (TSA), Columbia agar with colistin and nalidixic acid with 5% Sheep Blood (CNA) Agar, MacConkey Agar, Sabouraud Agar, Gardnerella Agar and Chocolate Agar in an anaerobic glove box (DW Scientific Anaerobic Workstation). The plates were incubated at 37°C for 24-48 hours in aerobic conditions, except for Gardnerella Agar plates which were placed in both a 5% CO_2_ incubator and anaerobiosis (two plates were seeded for each sample) and Chocolate agar which was incubated under anaerobic conditions. Anaerobiosis was created by using an anaerobic generator sachet (BD GasPak™) in the related jar. Specifically, approximately 1.5 mL of each sample was collected using a disposable Pasteur and subsequently sown on the selected media and then incubated at 37°C for 24-48 hours in aerobic, microaerobic or anaerobic conditions, as just described. In addition, about 2.5 mL of BHI were taken with the use of a sterile Pasteur and inoculated into a liquid enrichment broth, Fluid Thioglycollate Medium (FTM),that supports the growth of anaerobic bacteria without the use of an anaerobic chamber. The FTM liquid medium thus obtained was, together with the BHI medium, incubated at 37°C for 24 hours. Microbial growth was evaluated initially at 24 hours and subsequently at 48 hours after inoculating and diluting the previously incubated BHI medium on TSA, CNA Agar, MacConkey Agar and Sabouraud Agar. After 24 hours, the previously incubated FTM enrichment medium was inoculated and diluted on a solid Schaedler Agar medium and incubated at 37°C for 24-48 under anaerobic conditions as previously specified. Once the incubation period in anaerobic jars was over, the microbial growth of all prepared plates was evaluated. *Lactobacillus* was cultured on Gardnerella Agar and TSA in anaerobiosis conditions after 24 and 72 hours.

### 2.3 Phenotypical identification of microorganisms

The microorganisms were identified by MALDI-TOF MS (Bruken Daltonics, Bremen, Germany). In particular, it permits identification of organisms by species-specific profiles of peptide and protein masses. Specifically, a pure colony was taken from the culture plate using a wooden stick and transferred onto a MALDI-TOF MS target plate consisting of 96 spots. The spot on the target plate was then overlaid with 1-2 μL of matrix. Alternatively, when the direct spot test failed, the bacterial cells were coated with formic acid/acetonitrile before adding to the matrix. Fungal cells were treated with ethanol and formic acid/acetonitrile on the target plate before matrix overlay. This procedure was referred to as “On Target Lysis”. The matrix was applied within a short time to avoid oxidation of the sample on the target plate. After a short period of drying at room temperature, the plate was placed in the ion chamber of the mass spectrometer for analysis. The automatically generated mass spectrum was compared to a database of mass spectra using its software, resulting in the identification of the microorganism.

### 2.4 Statistical analysis

Data are presented as frequency (percentages). Comparisons between groups for categorical variables were performed with the chi-square test or with Fisher’s exact test as appropriate. Continuity correction was applied where necessary. For all analyses, the significance level was set at α = 0,05. All analyses were performed using the statistical software R, version 4.0.3.

## 3 Results

Features of the patients enrolled in the study including anthropometric characteristics, lifestyle related factors, hormonal values, causes and years of infertility and numbers of previous embryo transfer was reported in **Table 1**. Biochemical screening, microbiological and genetic tests and strumental exams pre IVF with relative results was listed in **Tables 2**, **3**.

**Table 1:** Characteristics of patients included in the study.

Characteristics	Mean ± SD	Prevalence (%)
**BMI**	**25,18 ± 1,06**	
**Smoke**		**32/93 (34,41%)**
**AMH**	**2,65 ± 0,41**	
**TSH**	**1,87 ± 0,29**	
**Estradiol (during embryo transfer cycle)**	**177,38 ± 184,55**	
**Triggering estradiol (during controlled ovarian stimulation)**	**1441,13 ± 253,85**	
**Progesterone pre-transfer**	**65,60 ± 43,77**	
**Years of infertility**	**4,51 ± 7,77**	
**Cause of infertility**		**Idiopathic 17/93 (18,28%); male 36/93 (38,71%); maternal age 12/93 (12,90%); combined 8/93(8,60%); tubal 7/93 (7,53%); dor 4/93 (4,30%); uterine 4/93 (4,30%); ovulatory 3/93 (3,22%); AEH 1/93 (1,08%); endometriosis 1/93**
**N° previous transfer**	**1,46 ± 1,41**	

Data are presented as mean ± standard deviation for continuous traits and prevalence (%). n, number of individuals; SD, standard deviation. BMI, Body mass index; AMH, Mullerian Inhbiting hormone; TSH, Thyroid Stimulating Hormone.

**Table 2:** Analysis or exams of patients included in the study before undergoing to IVF.

Analysis/exams	Negative/normal	Positive/abnormal
**Autoimmunity**	**Negative 90/93 (96,77%)**	**Positive 3/93 (3,23%)**
**Thrombophilia**	**Not tested 35/93 (37,63%)** **Negative 11/93 (11,83%)**	**Positive 47/93 (50,54%)**
Microbiology tests*	**Negative (75/93) (80,65%)**	**Positive (18/93) (19,35%)** **(Mixed flora 5/18; *Escherichia coli* 5/18; *Gardnerella vaginalis* 2/18; *Klebsiella pneumoniae* 2/18; *Candida albicans* 2/18; *Ureaplasma spp* 1/18; *Enterococcus faecalis* 1/18)**
**Hysteroscopy**	**Normal uterine cavity 67/93 (72,04%)**	**Intrauterine abnormalities 26/93 (27,96%)** **(polypectomy 16/26; fundal cuts 5/26 metroplasty 3/26; synechiolysis 1/26; unicorn uterus 1/26)** Endometritis 6/93 (6,45%)**

*cultural and molecular diagnostics analysis.

**Patients diagnosed with endometritis, by hysteroscopy exam and histological analysis, underwent a cycle of antibiotic therapy with doxycycline 100 mg twice a day for 14 days.

Data are presented as prevalence (%).

**Table 3:** Analysis/exams pre-*In vitro* Fertilization techniques in both groups.

Analysis/exams	Group A (N=35)	Group B (N=58)
**Vaginal-cervical swabs (positive)**	**8/35 (22,86%)**	**10/58 (17.24%)**
**Endometritis**	**2/35 (5,71%)**	**4/58 (6,90%)**
**Normal uterine cavity**	**24/35 (68,57%)**	**42/58 (72,41%)**
**Autoimmunity (positive)**	**1/35 (2,85%)**	**1/58 (1,72%)**
**Thrombophilia (positive)**	**15/35 (42,86%)**	**31/58 (53,45%)**
**Progesterone <9.2ng/mL**	**1/35 (2,86%)**	**4/58 (6,90%)**

Group A (pregnant patients) and Group B (no pregnant patients).

### 3.1 Culturomics results

**Figure 2:**
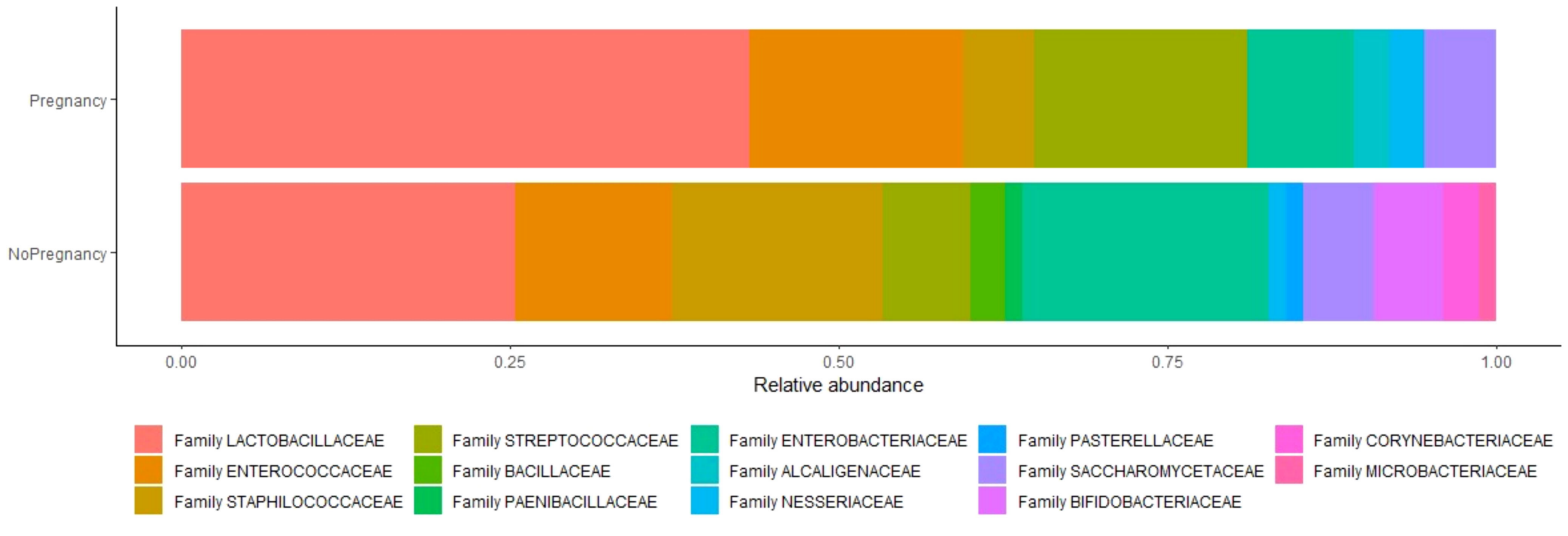
Relative abundance of species between two analyzed groups. Relative abundance is the percent composition of an organism of a particular kind relative to the total number of organisms in the area.

Sixtyeight infertile women (74%) resulted positive in the culture investigation for one or more bacterial species, while the remaining 25 patients (26%) showed no microbial development. A total of 136 pure colonies were subjected to MALDI TOF spectrometric analysis, through which 43 different taxonomic species were identified with an accurate score ≥ 2.0. Endometrial microbiological analysis allowed isolating up to 4 different phyla, and 14 different families and genera (**Table 4**, **Figure 2**).

**Table 4:** Species revealed from culturomics in all patients included in the study.

Species	Phylum	Family	Genus	Group A (%)	Group B (%)
***Alcaligenes faecalis***	**Proteobacteria**	**Alcaligenaceae**	**Alcaligenes**	**3,7**	**0**
***Bacillus halosaccharovans***	**Firmicutes**	**Bacillaceae**	**Bacillus**	**0**	**2,45**
***Bacillus simplex***	**Firmicutes**	**Bacillaceae**	**Bacillus**	**0**	**2,45**
***Bifidobacterium scardovii***	**Actinobacteria**	**Bifidobacteriaceae**	**Bifidobacterium**	**0**	**2,45**
***Candida albicans***	**Ascomycota**	**Saccharomycetaceae**	**Candida**	**0**	**4,9**
***Candida glabrata***	**Ascomycota**	**Saccharomycetaceae**	**Candida**	**3,7**	**0**
***Candida krusei***	**Ascomycota**	**Saccharomycetaceae**	**Candida**	**3,7**	**0**
***Candida lusitaniae***	**Ascomycota**	**Saccharomycetaceae**	**Candida**	**0**	**2,4**
***Candida parapsilosis***	**Ascomycota**	**Saccharomycetaceae**	**Candida**	**0**	**2,4**
***Citrobacter koseri***	**Proteobacteria**	**Enterobacteriaceae**	**Citrobacter**	**0**	**12,2**
***Corynebacterium spp***	**Actinobacteria**	**Corynebacyeriaceae**	**Corynebacterium**	**0**	**2,45**
***Corynebacterium coyleae***	**Actinobacteria**	**Corynebacyeriaceae**	**Corynebacterium**	**0**	**2,25**
***Enterobacter kobei***	**Proteobacteria**	**Enterobacteriaceae**	**Enterobacter**	**8,3**	**12,2**
***Enterococcus faecalis***	**Firmicutes**	**Enterococcaceae**	**Enterococcus**	**16,64**	**11,45**
***Escherichia coli***	**Firmicutes**	**Enterococcaceae**	**Escherichia**	**5,56**	**10,45**
***Gardnerella vaginalis***	**Actinobacteria**	**Bifidobacteriaceae**	**Gardnerella**	**0**	**7,35**
***Haemophilus haemolyticus***	**Proteobacteria**	**Pasteurellaceae**	**Haemophilus**	**0**	**2,4**
***Klebsiella pneumoniae***	**Proteobacteria**	**Enterobacteriaceae**	**Klebsiella**	**2,8**	**9,6**
***Lactobacillus crispatus***	**Firmicutes**	**Lactobacillaceae**	**Lactobacillus**	**11,1**	**9,08**
***Lactobacillus fermentum***	**Firmicutes**	**Lactobacillaceae**	**Lactobacillus**	**3,7**	**2,27**
***Lactobacillus gasseri***	**Firmicutes**	**Lactobacillaceae**	**Lactobacillus**	**18,5**	**11,35**
***Lactobacillus iners***	**Firmicutes**	**Lactobacillaceae**	**Lactobacillus**	**7,4**	**4,54**
***Lactobacillus jensenii***	**Firmicutes**	**Lactobacillaceae**	**Lactobacillus**	**11,1**	**4,54**
***Lactobacillus jonsonii***	**Firmicutes**	**Lactobacillaceae**	**Lactobacillus**	**3,7**	**2,27**
***Lactobacillus paracasei***	**Firmicutes**	**Lactobacillaceae**	**Lactobacillus**	**7,4**	**6,81**
***Lactobacillus rhamnosus***	**Firmicutes**	**Lactobacillaceae**	**Lactobacillus**	**7,4**	**4,54**
***Microbacterium maritypicum***	**Actinobacteria**	**Microbacteriaceae**	**Microbacterium**	**0**	**4,54**
***Neisseria subflava***	**Proteobacteria**	**Neisseriaceae**	**Neisseria**	**3,7**	**2,4**
***Paenibacillus glucanolyticus***	**Firmicutes**	**Paenibacillaceae**	**Paenibacillus**	**0**	**2,4**
***Paenibacillus spp***	**Firmicutes**	**Paenibacillaceae**	**Paenibacillus**	**0**	**2,4**
***Staphylococcus aureus***	**Firmicutes**	**Staphylococcaceae**	**Staphylococcus**	**0**	**2,43**
***Staphylococcus capitis***	**Firmicutes**	**Staphylococcaceae**	**Staphylococcus**	**0**	**2,43**
***Staphylococcus epidermidis***	**Firmicutes**	**Staphylococcaceae**	**Staphylococcus**	**7,4**	**17,01**
***Staphylococcus hominis***	**Firmicutes**	**Staphylococcaceae**	**Staphylococcus**	**0**	**2,43**
***Staphylococcus pasteuri***	**Firmicutes**	**Staphylococcaceae**	**Staphylococcus**	**0**	**2,43**
***Staphylococcus warneri***	**Firmicutes**	**Staphylococcaceae**	**Staphylococcus**	**0**	**2,43**
***Streptococcus agalactiae***	**Firmicutes**	**Streptococcaceae**	**Streptococcus**	**0**	**2,44**
***Streptococcus anginosus***	**Firmicutes**	**Streptococcaceae**	**Streptococcus**	**7,4**	**4,88**
***Streptococcus mitis***	**Firmicutes**	**Streptococcaceae**	**Streptococcus**	**3,7**	**2,44**
***Streptococcus oralis***	**Firmicutes**	**Streptococcaceae**	**Streptococcus**	**7,4**	**2,44**
***Streptococcus salivarius***	**Firmicutes**	**Streptococcaceae**	**Streptococcus**	**3,7**	**0**
***Streptococcus urinalis***	**Firmicutes**	**Streptococcaceae**	**Streptococcus**	**0**	**2,44**
***Streptococcus vestibularis***	**Firmicutes**	**Streptococcaceae**	**Streptococcus**	**0**	**2,44**

Group A (pregnant patients) and Group B (no pregnant patients).

Following the evaluation of the bacterial isolates, 47 infertile women (69,1%) were positive only for Gram-positive bacteria, 15 infertile women (22%) showed positivity for both Gram-positive and Gram- negative bacteria simultaneously, while the endometrium of 6 women (8,9%) had microbial colonization sustained exclusively by Gram-negative bacteria.

However, the results of the microbiological culture demonstrated that the uterine microbiota of the patients included in the study mainly consisted of bacteria belonging to the following phyla: *Firmicutes* (87,76%), *Proteobacteria* (27,94%), *Actinobacteria* (10,29%) and *Ascomycota* (8,82%).

The phylum *Firmicutes* was the most abundant operative taxonomic unit, isolated in 59 of 68 infertile women (87,35%). In particular, the genera revealed were *Lactobacillus spp* (51,47%), *Enterobacter spp* (25%), *Enterococcus* spp. (22,05%), *Staphylococcus* spp.(20,58%), *Streptococcus* spp. (16,17%), *Saccharomyces* spp. (8,82%), *Bifidobacteria* spp. (5,88%), *Neisseria* spp. (2,94%), *Corynebacterium* spp. (2,94%), *Alcaligenes* spp. (1,47%), *Bacillus* spp. (2,94%), *Paenibacillus* spp. (1,47%), *Haemophilus* spp. (1,47%) and *Microbacterium* spp. (1,47%). Therefore, from the cultural analysis it is evident that the greatest number of infertile women (35) had a clear prevalence of the lactobacillary population in the uterine cavity. In particular, the *Lactobacillus* species isolated by mass spectrometry with MALDI-TOF technology were the following: *Lactobacillus gasseri*, *Lactobacillus crispatus*, *Lactobacillus jensenii, Lactobacillus iners, Lactobacillus rhamnosus, Lactobacillus fermentum, Lactobacillus paracasei and Lactobacillus johnsonii*.

### 3.2 Comparison of pregnant and non-pregnant women

Taking into account patients who achieved clinical pregnancy independently from the age (35/93, 37,6%), 27 patients (39,7%) showed endometrial bacterial colonization, while only 8 patients showed no microbial development (33,3%). In terms of reproductive outcome, we did not detect significant differences between infertile women who tested positive for microbiology and those with no microbial development.

The microbial species present in infertile women who obtained a clinical pregnancy (Group A) were compared with the isolated species in those with an unfavorable IVF outcome (Group B).

Results showed a significant difference in *Lactobacillus* spp. isolation in patients of Group A with respect to implantation failure (37% vs 5% p=0,05) (**Table 5**). In addition, the presence of the following families was associated with a poor prognosis of the IVF technique after blastocyst transfer: *Staphylococcaceae* (8% vs 35%, p<0,05); *Enterobacteriaceae* (60% vs 100%, p<0,001) (**Table 5**).

**Table 5:** Comparison between Group A (pregnant patients) and Group B (no pregnant patients).

Phylum	Family	Group A %	Group B %	P-value
**Firmicutes**	**Lactobacillaceae**	**37**	**5**	**0.05**
**Firmicutes**	**Staphylococcaceae**	**8**	**35**	**0.034**
**Proteobacteria**	**Enterobacteriaceae**	**60**	**100**	**<0.001**
**Actinobacteria**	**Bifidobacteriace Corynebacteriace** **Microbacteriaceae**	**0**	**17**	**0,037**

Data are presented as relative prevalence among the families of the phylum (%). *Relative prevalence with respect to the distribution of the Phylum.

However, although the uterine microbiota of the two different groups was found to be very similar in terms of taxonomic variety, the Phylum *Actinobacteria* including *Bifidobacteriaceae, Corynebacteriaceae* and *Microbacteriaceae* families was exclusively expressed in the group of women who did not achieve a clinical pregnancy (Group B, P=0,037) (**Table 5**). Specific species identified were *Bacillus simplex, Bacillus halosaccharovorans, Paenibacillus glucanolyticus, Haemophilus haemolyticus, Bifidobacteria scardovii, Gardnerella vaginalis, Corynebacterium coyleae and Microbacterium maritypicum*.

In addition, MALDI TOF analysis also revealed the presence of fungi such as *Candida albicans, Candida glabrata, Candida krusei, Candida parapsilosis and Candida lusitaniae*. The two groups include a different identification of species of *Ascomycota*: Group A was positive for *Candida krusei* and *Candida glabrata*; Group B for *Candida albicans, Candida parapsilosis* and *Candida lusitanie*.

## 4 Discussion

Focusing on the results of this study, the percentage of patients positive to the culture investigation for one or more species stands out in the foreground. Indeed, 74% of patients is a data that definitely open the discussion about the endometrial investigation prior to embryo transfer. Significant results of this study demonstrate a positive impact of *Lactobacillus* spp. on ongoing pregnancy rate. At the same time, several findings including the different expression of some families (*Staphylococcaceae, Enterobacteriaceae*) between the two analyzed groups and the exclusively expression of the Phylum *Actinobacteria* in the group of women who did not achieve a clinical pregnancy demonstrate the

potential negative impact of disbiotic microbiota on IVF outcome.

Many studies have examined the endometrial microbiome on IVF outcome. However, most of these have been performed applying a metagenomics approach (14). The use of new genomic sequencing technologies applied to the study of the uterine microbiota has limitations as the latter is considered a low biomass microbiome. Culture studies of the endometrial microbiota are rare. Only very few studies describe the use of culture techniques on endometrial samples, and almost all are designed to reveal specific opportunistic bacteria, such as *Escherichia coli* and *Gardnerella vaginalis*.

In this study the endometrial microbiota was analyzed by a culturomics method to test its feasibility in the clinical IVF procedure. Each sample was seeded on solid and liquid media. The bacterial population present in the samples was evaluated primarily by growth on solid media. Enrichment broths were considered only in cases where no colonies were obtained on solid plates, to recover even minority species.

The endometrium is not a sterile tissue and, in line with our work, populations of endometrial-resident microorganisms have also been observed in the literature (5). The endometrial microbiological evaluation performed in the candidate patients for study allowed the delineation of two populations, which differed from each other according to the success or otherwise of the IVF technique. In contrast to Franasiak et al., 2016 but in agreement with Moreno et al., 2016 the results of that study suggested that a *Lactobacillus-*dominant endometrial microbiota was associated with a significant increase in the pregnancy rate (4, 10, 15). In addition, it has been previously demonstrated that a non-*Lactobacillus*-dominated microbiota was correlated with significant decreases in implantation, pregnancy, ongoing pregnancy and live birth rates. The *Lactobacillus* species improve the IVF outcome thanks to lactic acid secretion, which enhances suppression of pathogenic germs by promoting an acid environment in the vagina (16). These results suggest that the genus *Lactobacillus* could be a biomarker of endometrial microflora for IVF treatment success.

In addition, *Staphylococcus* spp. was significantly prevalent in samples deriving from women with implantation failure. Urogenital bacterial infections have lately correlated especially well with male infertility (17, 18). In fact, it has been shown that *Staphylococcus aureus* is able to compromise sperm motility and viability and thus IVF outcome as also demonstrated by a low pregnancy rate among couples with positive semen cultures (19). In particular, the endometrial bacterial colonization could interact with the endometrium and/or alter endometrial expression of cytokines necessary for successful blastocyst development and implantation. A deficiency of interleukin (IL)-11, leukemia inhibitory factor (LIF), and transforming growth factor β (TGF-β1) leads to implantation failure and abnormalities in placental formation (20). Furthermore, cytokine expression is modulated by Toll-like receptors (TLRs), which are highly expressed in the reproductive tract and are capable of recognizing molecular patterns, such as lipopolysaccharide (LPS) and Peptidoglycan (21). The impact of TLRs on reproductive outcomes has been extensively studied by Koga, K et al., 2014, and it is possible to hypothesize that fluctuations in microbial communities induce a host immune response in the genital tract, increasing the production of proinflammatory cytokines that could compromise the implantation of the embryo and determine unfavorable reproductive outcomes during the IVF cycles. In addition, a possible consequence of inflammation status could be promotion of the development of a progesterone-resistant endometrium (22, 23). Definitely, the “freeze all” strategy has been recognized as an efficient way to manage some IVF cycles including storing embryos for transfer in subsequent cycles after pre-implantation testing or to store surplus embryos (23, 24).

Although further studies are needed to better understand the role of endometrial bacteria in reproductive function, these preliminary results suggest an important role for microbial communities in embryo implantation and pregnancy establishment, and also support the use of transfer catheters of embryos associated with scrupulous microbiological culture investigation to test the endometrial microbiota.

The two technologies used to test the endometrial microbial communities, NGS and culturomics, are compared in **Table 6**. NGS can boast of being a fast method, of providing data in a quantitative measure and with greater sensitivity (25). Conversely, the biggest disadvantage of NGS is the generation of chimeric sequences and sequencing errors. Another limitation lies in adequately exploring low biomass settings, an advantageous feature we instead find in culturomics (26). Among the other advantages of culturomics we mention the possibility of identifying vital bacteria and fungi as well as the possibility of plate cultures in anaerobic and aerobic conditions (27). Even if culturomics provides additional information, this information is only available in qualitative terms, where we recall instead that sequencing is also able to provide a representation of the quantitative composition of the microbiota. Clearly, this study demonstrates that culture-based testing is an efficient first-level method to define microbiota in clinical practice.

**Table 6:** Comparison between NGS and culturomics.

Features	NGS (Next Generation Sequencing)	Culturomics
*Identification of viable bacteria and fungi to species level*	no	yes
*Detection of microorganisms at a very low biomass level*	no	yes
*Taxonomic resolution*	yes	yes
*Distinguish between live bacteria and pieces of nucleic acids*	no	yes
*Microbiome analysis without culture*	yes	no
*Quantitative representation of the microbiome*	yes	no
*Culturing under both anaerobic and aerobic conditions*	no	yes
*Generation of chimeric sequences*	yes	no
*DNA/RNA contamination*	yes	yes
*High workload*	no	yes
*Shorten turnaround time*	yes	no

A limitation of this study is the possible contamination of the microbiota population in the uterus with the vaginal microbiome. Although a strategy to isolate the transfer catheter was applied as described above it is not possible to exclude completely a contamination from the cervix.

In conclusion, a eubiotic endometrial microbiota could be considered as a permissive microbial community for ongoing pregnancy, regardless of the presence of minimal pathogenic bacteria. Finally, the study of endometrial microbiota could be a future means of improving reproductive outcomes in infertile patients. Therefore, standardized protocols and larger patient cohorts are needed for studies to be comparable and help better understand uterine microbiota.

## Data availability statement

The raw data supporting the conclusions of this article will be made available by the authors, without undue reservation.

## Ethics statement

Ethical review and approval was not required for the study on human participants in accordance with the local legislation and institutional requirements. The patients/participants provided their written informed consent to participate in this study.

## Author contributions

FC and CC contributed to conception and design of the study. FB organized the sampling. VM organized the database. DP performed the statistical analysis. FC wrote the first draft of the manuscript. RDG and AC edited and reviewed the manuscript. RP, MRC, IS, and CA revised manuscript. All authors contributed to manuscript revision, read, and approved the submitted version.
