# Corrigendum: Endometrial microbiota profile in *in-vitro* fertilization (IVF) patients by culturomics-based analysis

**DOI:** 10.3389/fendo.2024.1362947

**Published:** 2024-01-23

**Authors:** Federica Cariati, Consolata Carotenuto, Francesca Bagnulo, Daniela Pacella, Vincenzo Marrone, Rossella Paolillo, Maria Rosaria Catania, Raffaella Di Girolamo, Alessandro Conforti, Ida Strina, Carlo Alviggi

**Affiliations:** ^1^ Department of Public Health, School of Medicine, University of Naples Federico II, Naples, Italy; ^2^ Department of Molecular Medicine and Medical Biotechnology, University of Naples Federico II, Naples, Italy; ^3^ Department of Neuroscience, Reproductive Science and Odontostomatology, University of Naples Federico II, Naples, Italy

**Keywords:** microbiota, endometrium, embryo transfer, IVF, MALDI

**Text Correction**

In the published article, there was an error. In methods section of the abstract, an incorrect study period was indicated. However, the correct one was already reported in the in main body of the manuscript (Material and methods section).

A correction has been made to abstract, methods. This sentence previously stated: “A prospective cohort study was performed at the University of Naples from June 2022 to December 2022”.

The corrected sentence appears below:

“A prospective cohort study was performed at the University of Naples from October 2022 and February 2023”.

**Error in Figure/Table**

In the published article, **Figure 2** and **Table 5** reported errors. In details, in **Figure 2A** a color label association error was made. **Table 5** showed an error in data on Firmicutes/Lactobacillaceae presented as absolute value and not as a percentage. The corrected **Figure 2** and **Table 5** and its caption appear below.

**Figure 2:**
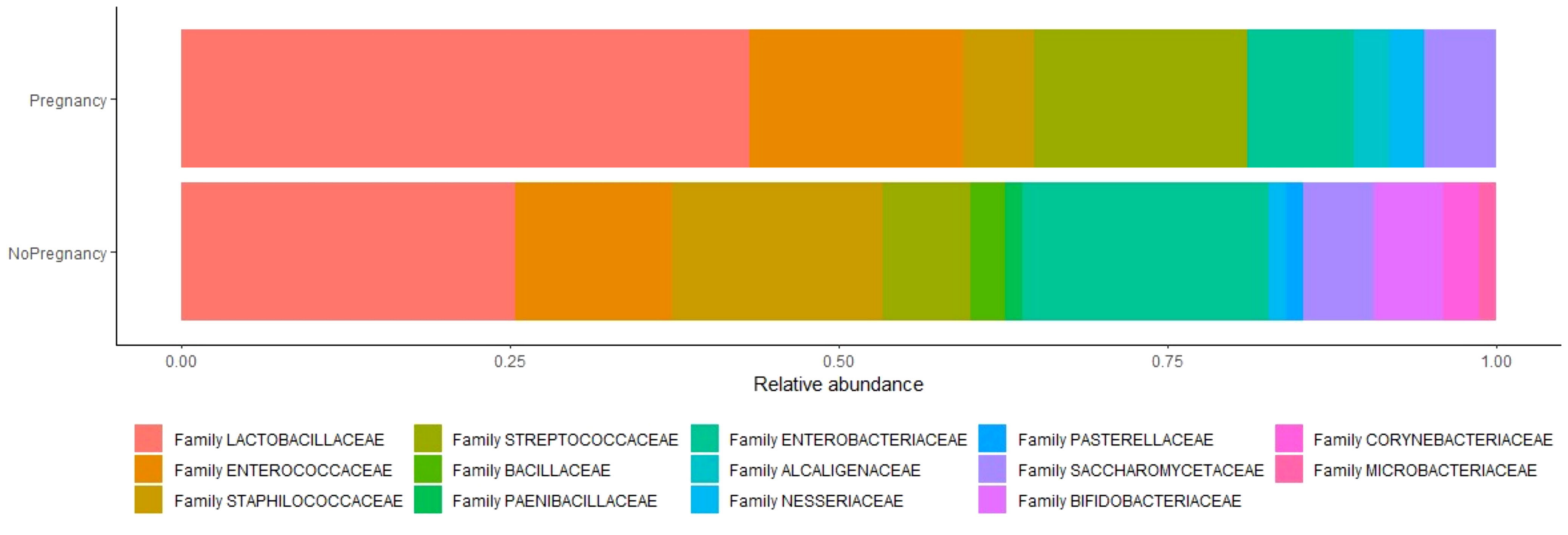
Relative abundance of species between two analyzed groups. Relative abundance is the percent composition of an organism of a particular kind relative to the total number of organisms in the area.

**Table 5:** Comparison between Group A (pregnant patients) and Group B (no pregnant patients).

Phylum	Family	Group A %	Group B %	P-value
**Firmicutes**	**Lactobacillaceae**	**15**	**40**	**0.05**
**Firmicutes**	**Staphilococcaceae**	**8**	**35**	**0.034**
**Proteobacteria**	**Enterobacteriaceae**	**60**	**100**	**<0.001**
**Actinobacteria**	**Bifidobacteriaceae** **Corynebacteriaceae** **Microbacteriaceae**	**0**	17*	**0.037**

Data are presented as relative prevalence among the families of the phylum (%). *Relative prevalence with respect to the distribution of the Phylum.

**Error in Table Legend**

In the published article, there was an error in the legend for **Table 5** as published.

The legend “ Comparison between Group A (pregnant patients) and Group B (no pregnant patients)” does not properly match the contents.

The corrected legend appears below:

Comparison between Group A (pregnant patients) and Group B (no pregnant patients). Data are presented as relative prevalence among the families of the phylum (%). *Relative prevalence with respect to the distribution of the Phylum.

The authors apologize for these errors and state that they does not change the scientific conclusions of the article in any way.

